# High-Sensitivity C-Reactive Protein is Related to Central Obesity and the Number of Metabolic Syndrome Components in Jamaican Young Adults

**DOI:** 10.3389/fcvm.2014.00012

**Published:** 2014-12-16

**Authors:** Nadia R. Bennett, Trevor S. Ferguson, Franklyn I. Bennett, Marshall K. Tulloch-Reid, Novie O. M. Younger-Coleman, Maria D. Jackson, Maureen E. Samms-Vaughan, Rainford J. Wilks

**Affiliations:** ^1^Epidemiology Research Unit, Tropical Medicine Research Institute, The University of the West Indies Mona, Kingston, Jamaica; ^2^Department of Pathology, The University of the West Indies Mona, Kingston, Jamaica; ^3^Department of Community Health and Psychiatry, The University of the West Indies Mona, Kingston, Jamaica; ^4^Department of Child and Adolescent Health, The University of the West Indies Mona, Kingston, Jamaica

**Keywords:** high-sensitivity C-reactive protein, cardiovascular disease, metabolic syndrome, young adults, Jamaica, Caribbean, Black

## Abstract

**Background:** High-sensitivity C-reactive protein (hsCRP) has been shown to predict cardiovascular disease (CVD) endpoints and is associated with CVD risk factors and the metabolic syndrome. This study evaluated the association between hsCRP and CVD risk factors among Afro-Caribbean young adults in Jamaica.

**Methods:** We conducted a cross-sectional analysis of data from the Jamaica 1986 Birth Cohort Study. Data were collected between 2005 and 2007 when participants were 18–20 years old. All participants completed an interviewer administered questionnaire and had anthropometric and blood pressure (BP) measurements performed. Fasting blood samples were collected for measurement of glucose, lipids, and hsCRP. Logistic regression models were used to identify factors independently associated with high hsCRP.

**Results:** Analyses included 342 men and 404 women with mean age 18.8 ± 0.6 years. Approximately 15% of the participants had high risk hsCRP (>3 mg/L), with a higher prevalence among women (20 vs. 9%; *p* < 0.001). The prevalence of elevated hsCRP increased with body mass index category, high waist circumference (WC), high triglycerides, low high density lipoprotein, and lower parental education among women, but only for high WC and lower parental education among men. In logistic regression models controlling for sex and parental education, high WC was associated with significantly higher odds of high hsCRP (OR 7.8, 95% CI 4.8–12.9, *p* < 0.001). In a similar model, high hsCRP was also associated with the number of metabolic syndrome components. Compared to participants with no metabolic syndrome component, having one metabolic syndrome component was associated with a twofold higher odds of high hsCRP (OR 2.2, 95% CI 1.3–3.8, *p* = 0.005), while having three components was associated with a 14-fold higher odds of high hsCRP (OR 13.5, 95% CI 2.4–76.0, *p* < 0.001).

**Conclusion:** High hsCRP is common among Jamaican young adults and is strongly associated with central obesity and the number of metabolic syndrome components.

## Introduction

Almost 50% of persons with acute myocardial infarction in the USA have no overt evidence of hyperlipidemia, and 15–20% have none of the traditional cardiovascular disease (CVD) risk factors (1, 2). As a result, there has been considerable interest in identifying risk markers that will refine and improve the clinician’s ability to predict CVD (3). Inflammation is now regarded as one factor that might explain this phenomenon. It is generally accepted that inflammation plays an active role in the pathogenesis of CVD and is a possible trigger of CVD events (4, 5). Several clinical studies have demonstrated that chronic inflammation is a predictor of future CVD events and that biomarkers of inflammation can improve risk prediction in CVD (4–6). In adults, inflammation is an independent risk factor for both the metabolic syndrome and CVD (7, 8).

One increasingly popular marker of inflammation is C-reactive protein (CRP), an acute phase protein, which is markedly elevated in inflammatory conditions such as infections, and is also elevated in CVD, but in much lower concentrations (9–12). The development of assays capable of measuring mild elevation of CRP, termed high-sensitivity C-reactive protein (hsCRP), resulted in the wide-scale use of this biomarker in the evaluation of CVD risk (10). High-sensitivity CRP has been shown to predict the risk of CVD endpoints such as myocardial infarction, stroke, and CVD death independent of other traditional risk factors for CVD (2, 4, 5). In addition, some studies have shown associations between high hsCRP and socioeconomic status (SES) (13–15).

Most studies of the relationship between hsCRP and CVD have been done in middle-aged or older persons; however, associations between hsCRP and CVD risk factors among adolescents and young adults have been reported in a few studies (16–18). In children and adolescents, even low-grade systemic inflammation has been shown to be associated with the metabolic syndrome (19, 20). To our knowledge, no study has evaluated the associations between hsCRP and CVD risk in young adults from the Caribbean, where a high burden of CVD risk factors have been demonstrated (21). We therefore sought to evaluate the relationship between hsCRP levels and CVD risk factors among participants of the Jamaica 1986 Birth Cohort Study. We chose hsCRP as the inflammatory biomarker because of the large body of data supporting its usefulness as a marker of CVD risk and because laboratory assays are available for its use in current clinical practice.

We hypothesized that high hsCRP levels are associated with individual CVD risk factors and with clustering of CVD risk factors as seen in the metabolic syndrome. Specifically, we aimed to (1) estimate the proportion of persons with high risk hsCRP; (2) evaluate the relationship between elevated hsCRP and traditional CVD risk factors [blood pressure (BP), glucose, obesity, dyslipidemia]; and (3) evaluate whether elevated hsCRP is associated with the clustering of multiple CVD risk factors expressed as the number of metabolic syndrome components. We also explored whether high hsCRP was associated with SES (using parental education level).

## Materials and Methods

### Study design

This study was a cross-sectional analysis from the Jamaica 1986 Birth Cohort Study (22). Details of the study design, follow up, and recruitment procedures have been previously published (22, 23). In the third follow up round of the cohort, 902 participants, aged 18–20 years were seen between March 2005 and February 2007. The study was approved by the University of the West Indies, Faculty of Medical Sciences Ethics Committee, and all participants provided written informed consent prior to evaluation.

### Measurements and definitions

Trained research nurses administered questionnaires during face to face interviews in order to obtain data on personal and family medical history, SES, and physical activity levels. The nurses also obtained BP and anthropometric measurement (weight, height, waist, and hip circumference) using standard techniques. A fasting blood sample was collected for glucose, lipid levels, and hsCRP.

Blood pressure was measured using a mercury sphygmomanometer following standardized procedures developed for the International Collaborative Study of Hypertension in Blacks (24). Waist circumference (WC) was measured using a non-stretchable nylon tape. Height was measured using a portable stadiometer and weight was measured using a portable digital scale. Elevated BP was defined as systolic BP ≥120 mmHg or diastolic BP ≥80 mmHg, incorporating the prehypertension and hypertension categories, as defined by the Seventh Report of the Joint National Committee on Prevention, Detection, Evaluation, and Treatment of High BP (JNC 7) (25). Body mass index (BMI) was calculated as weight in kilograms divided by the square of height in meters and the World Health Organization classification used to define overweight as BMI of 25.0–29.99 and obesity as BMI ≥30.0 kg/m^2^ (26). Central obesity, high fasting glucose, high triglycerides, and low high density lipoprotein (HDL) cholesterol were defined using the metabolic syndrome cut points recommended by the 2009 Consensus Criteria by the International Diabetes Federation, American Heart Association, and others (27). The specific cut points were: central obesity ≥94 cm for men and ≥80 cm for women; high fasting glucose, ≥5.6 mmol/L; high triglycerides, ≥1.7 mmol/L; low HDL <1.0 mmol/L in men and <1.3 mmol/L in women. For metabolic syndrome categorization, high BP was determined using cut points of ≥130 mmHg for systolic BP and ≥85 mmHg (27). Participants with any three of the five components were classified as having the metabolic syndrome as defined by the 2009 Consensus Criteria (27). High total cholesterol was defined as cholesterol ≥5.2 mmol/L and increased low density lipoprotein cholesterol (LDL) was defined as LDL ≥4.1 mmol/L in accordance with the recommendations from National Cholesterol Education Program Adult Treatment panel III criteria (ATP III) Expert Panel (28).

High-sensitivity CRP was measured from stored serum samples using the IMMULITE^®^ 1000 High-Sensitivity CRP enzyme immunoassay test kits (Siemens Medical Solution Diagnostics, Los Angeles CA, USA). Tests were performed in duplicate by a single laboratory technician using standard quality control procedures to ensure validity and reliability of test results. The test has an analytic reportable range of 0.3–100 mg/L. Where participants had values below the reference range, they were assigned a value of 0.3 mg/L. For analysis, the mean of the two values were used. Risk categories recommended by the American Heart Association and Center for Disease Control were used to categorize hsCRP as low risk (hsCRP <1 mg/L), moderate risk (hsCRP 1–3 mg/L) and high risk (hsCRP >3 mg/L) (8). For outcome analyses, we defined high hsCRP as hsCRP >3 mg/L. Participants with hsCRP >10 mg/L were excluded from the analyses as these levels may reflect infection or inflammatory disorder (8).

Socioeconomic status was defined as the highest level of education attained by either parent or guardian and categorized as: primary/all age, secondary, or tertiary. Persons who responded “do not know” to the questions on education of parents/guardian were treated as a fourth category. Leisure time physical activity levels were defined based on time spent playing sports or engaged in exercise (e.g., brisk walking). Participants who reported no leisure time physical activity were classified as having low physical activity level, participants reporting 3.5 h or less of physical activity per week as moderate of physical activity level, while those reporting more than 3.5 h of physical activity per week were classified as high physical activity level.

### Statistical analysis

Data were analyzed using Stata 12.1 (Stata Corp., College Station, TX, USA). Descriptive analyses included means and proportions for outcome and explanatory variables as appropriate. Where data were highly skewed, we reported medians and interquartile range. Means were compared using the *t*-test while difference in proportions were compared using the Pearson’s chi-squared test. For comparison of means across multiple categories, we used one-way analysis of variance or the Kruskal–Wallis test if there was evidence on unequal variance across the categories. As there was evidence of sex interaction in the relationship between some CVD risk factors and high hsCRP, we present sex-specific estimates for bivariate analyses. Logistic regression was used to identify factors independently associated with high hsCRP. All variables, which showed evidence of association (*p* < 0.05) in the bivariate analyses and those with an *a priori* hypothesized association, were sequentially added to the model and the likelihood ratio test used to identify variables, which significantly improved the model.

In order to assess whether hsCRP was associated with clustering of CVD risk factors, we created a variable based on the number of metabolic syndrome components present in participants (elevated BP, high fasting glucose, central obesity, low HDL, and high triglycerides). We then assessed whether the number of risk factors was significantly associated with high hsCRP.

Since our study tested two primary hypotheses, the significance level for the final models was adjusted to 0.025 using the Bonferroni method as recommended by Bender and Lange (29) and by Sedgwick (30).

Data on hsCRP was unavailable for 61 participants and 45 participants had hsCRP values >10 mg/L. Another 50 participants had missing values for at least one additional variable of interest. The final analysis was therefore limited to 746 participants with full data for the variables of interest.

## Results

Analyses included 342 men (46%) and 404 women (54%) with mean age 18.8 ± 0.6 years. Sex-specific characteristics of the study participants are shown in Table 1. Men had higher systolic BP, diastolic BP, fasting glucose, and triglycerides whereas women had higher mean total cholesterol, LDL, HDL, and hsCRP. In order to assess the potential impact of participants who were excluded from the analyses, we compared mean values of the characteristic shown in Table 1 (except for hsCRP) for included and excluded participants. There were no significant differences for participants included in the analysis compared to those excluded.

**Table 1:** **Summary statistics for participant characteristics by sex**.

Characteristic	Male (*n* = 342)	Female (*n* = 404)	*P*-value for male: female differences^a^
	**Mean ± SD**	**Mean ±SD**	
Age	18.8 ± 0.6	18.8 ± 0.6	0.188
Height (cm)	176.8 ± 6.5	163.4 ± 6.2	<0.001
Weight (kg)	70.8 ± 13.9	61.7 ± 14.4	<0.001
BMI (kg/m^2^)	22.6 ± 4.2	23.1 ± 5.2	0.200
Waist circumference (cm)	75.1 ± 10.9	73.3 ± 11.0	0.033
Systolic BP (mmHg)	114.0 ± 10.7	107.1 ± 8.7	<0.001
Diastolic BP (mmHg)	69.4 ± 10.2	66.9 ± 8.9	<0.001
Glucose (mmol/L)	4.7 ± 0.6	4.4 ± 0.4	<0.001
Total cholesterol (mmol/L)	4.2 ± 0.8	4.5 ± 0.9	<0.001
HDL (mmol/L)	1.1 ± 0.2	1.3 ± 0.3	<0.001
LDL (mmol/L)	2.7 ± 0.7	3.0 ± 0.8	<0.001
Triglycerides (mmol/L)	0.60 ± 0.3	0.55 ± 0.2	0.007
hsCRP (mg/L)	1.1 ± 1.5	1.8 ± 2.1	<0.001
	**Median (IQR)**	**Median (IQR)**	
^b^hsCRP (mg/L)	0.5 (0.9)	1.0 (2.0)	<0.001
	**Number (%)**	**Number (%)**	
hsCRP risk categories			<0.001
Low risk	235 (68.7)	208 (51.5)
Moderate risk	78 (22.8)	114 (28.5)	
High risk	29 (8.5)	81 (20.0)	
Highest parental SES			0.005
Tertiary education	73 (21.4)	114 (28.2)	
Secondary education	179 (52.3)	194 (48.0)	
Primary education	32 (9.4)	54 (13.4)	
Unknown	58 (17.0)	42 (10.4)	
Physical activity levels (PAL)			<0.001
High PAL	124 (36.3)	54 (13.4)	
Moderate PAL	155 (45.3)	165 (40.8)	
Low PAL	63 (18.4)	185 (45.8)	

*^a^*P*-values for means from *t*-tests comparing males and females while *p*-values for difference in proportions are from chi-squared tests again comparing males to females*.

*^b^The hsCRP distribution was skewed; hence, we also present median and interquartile range (defined as the difference between the 75th and 25th centiles)*.

*hsCRP, high sensitivity C-reactive protein; SES, socioeconomic status; PAL, physical activity levels; BMI, body mass index; BP, blood pressure; HDL, high density lipoprotein cholesterol; LDL, low density lipoprotein cholesterol. Total cholesterol includes LDL, HDL, and other lipoproteins*.

The proportion of participants with individual CVD risk factors and high hsCRP (>3 mg/L) is shown in the Supplementary Material (Table S1 in Supplementary Material). Approximately 15% of the participants had high risk hsCRP with women having a prevalence of 20% compared to 9% among men (*p* < 0.001). Approximately 21% of participants had elevated BP (BP ≥120/80 mmHg), 17% were overweight and another 7% obese; 14% of participants had high cholesterol and 46% had low HDL. Prevalence of elevated triglycerides and impaired fasting glucose was relatively low, 0.5 and 1.1%, respectively; 6% had high LDL cholesterol. Men were more likely to have elevated BP and fasting glucose, while women had a higher prevalence of all other CVD risk factors. High triglyceride was rare in both men and women.

Table 2 shows the proportion of participants with high hsCRP (>3 mg/L) by traditional CVD risk factor categories and SES (based on parental education level). Only central obesity and parental education were significantly associated with high hsCRP among men, but associations were also seen for BMI, low HDL, and high triglycerides among women. There was no significant association between high hsCRP and physical activity level (data not shown).

**Table 2:** **Proportion of participants with high risk hsCRP within categories of CVD risk factors and socioeconomic status (parental education level) for male and female participants**.

Variable	Male			Female		
	Number in category	High hsCRP number (%)	*P*-value	Number in category	High hsCRP number (%)	*P*-value^a^
BMI			0.111			<0.001
Underweight	23	1 (4.3)		58	6 (10.3)	
Normal weight	255	18 (7.1)		229	27 (11.8)	
Overweight	47	4 (8.5)		79	21 (26.6)	
Obese	17	6 (35.3)		38	27 (71.1)	
Elevated blood pressure			0.486			0.258
No	241	22 (9.1)		351	67 (19.1)	
Yes	101	7 (6.9)		53	14 (26.4)	
High WC			0.048			<0.001
No	326	24 (7.4)		312	34 (10.9)	
Yes	16	5 (31.3)		92	47 (51.1)	
High glucose^b^			0.274			0.293
No	245	18 (7.3)		372	72 (19.4)	
Yes	97	11 (11.3)		32	9 (28.2)	
High cholesterol			0.693			0.940
No	314	26 (8.2)		328	66 (20.1)	
Yes	28	3 (10.7)		76	17 (19.7)	
Low HDL			0.246			0.002
No	247	18 (7.3)		156	20 (12.8)	
Yes	95	11 (11.6)		248	61 (24.6)	
Increased LDL			0.877			0.460
No	332	28 (8.4)		366	75 (20.5)	
Yes	10	1 (10.0)		38	6 (15.8)	
Increased triglycerides^c^			0.606			0.044
No	271	24 (8.9)		325	58 (17.8)	
Yes	71	5 (7.0)		79	23 (29.1)	
Parental education level			0.005			0.049
Tertiary education	73	5 (6.8)		114	14 (12.3)	
Secondary education	179	15 (8.4)		194	42 (21.6)	
Primary/all age	32	8 (25.0)		54	14 (25.9)	
Unknown	58	1 (1.7)		42	11 (26.2)	

*^a^*P*-values are from proportion post-estimation Wald tests. Adjustment for multiple testing was not performed as these were considered preliminary exploratory analyses. If the Bonferroni correction for multiple testing were to be applied, the *p*-value for statistical significance would be 0.006*.

*^b^High glucose here is defined as upper quintile of glucose distribution*.

*^c^High triglyceride here is defined as upper quintile of triglyceride distribution*.

*hsCRP, high sensitivity C-reactive protein; BMI, body mass index; HDL, high density lipoprotein cholesterol; LDL, low density lipoprotein cholesterol*.

We also obtained mean values for CVD risk factors by hsCRP category for men and women. These data are shown in Table S2 in Supplementary Material. Among men, significant associations with hsCRP category were seen for BMI and WC only with persons in the high risk hsCRP category having higher mean BMI and WC. Among women, similar associations were seen for BMI and WC, but additionally there were significant associations for systolic BP, LDL, and triglycerides. Mean HDL was lower among women with high risk hsCRP.

**Figure 1:**
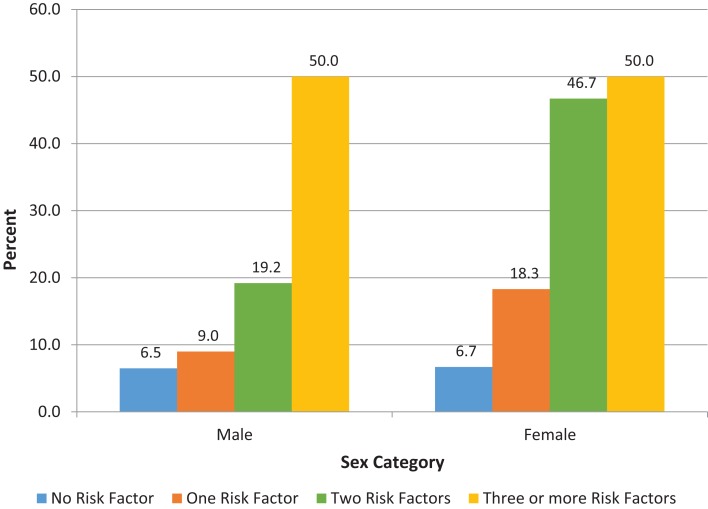
**Proportion (%) of participants with high hsCRP based on number of metabolic syndrome components for male and female participants**. The proportion of participants with high hsCRP increased with the number of the metabolic syndrome components for males (*p* = 0.025), and for females (*p* < 0.001). *P*-values were derived from chi-squared tests. Tests for trend were also significant: *p* = 0.011 for males; *p* < 0.001 for females. hsCRP, high sensitivity C-reactive protein.

In order to evaluate the association between high hsCRP and clustering of CVD risk factors, we computed a risk score based on the number of metabolic syndrome components that a participants had. We also evaluated the association between high hsCRP and the metabolic syndrome. Results for these analyses are shown in Figure 1 and Table S3 in Supplementary Material. Approximately 39% of participants had one of the five risk factors, 14% two risk factors, and 0.8% of participants had the metabolic syndrome. There was a statistically significant trend for higher prevalence of high hsCRP as the number of risk factors increased for both men and women (Figure 1). Mean and median hsCRP increased with the number of risk factors with a statistically significant test for trend (see Table S3 in Supplementary Material).

Results for the multiple logistic regression analyses, using high hsCRP as the outcome are shown in Table 3. BMI and WC were highly correlated (Pearson’s correlation coefficient, *r* = 0.81) therefore we opted to use WC as the measure of adiposity in our models. Model 1 assessed the relationship between high hsCRP (outcome variable) and individual CVD risk factors and SES. Only central obesity and parental education remained statistically significant correlates of hsCRP. Odds ratio for high hsCRP for those with central obesity compared to those without was 7.8 (95% CI 4.8–12.9, *p* < 0.001). Participants whose highest parental education level was in the primary/all-age category had a twofold higher odds of high hsCRP compared to those with tertiary level parental education (OR 2.47, 95% CI 1.17–5.20, *p* = 0.017). In the second model, we examined whether the number of metabolic syndrome components was associated with high hsCRP. After adjusting for sex and parental education, having one component was associated with a twofold increase in the odds of high hsCRP, while having three or more components (i.e., having the metabolic syndrome) was associated with a 14-fold increase in the odds of high hsCRP. There was some evidence of sex interaction in the relationship between hsCRP and education category (*p* = 0.049 for the interaction term for the “unknown” education category). We therefore also present sex-specific estimates for these models in Table S4 in Supplementary Material. The result from these models was generally similar to the un-stratified models, but estimates were less precise with wide confidence intervals.

**Table 3:** **Final multivariable models for high hsCRP individual CVD risk factors and number of metabolic syndrome components**.

Variable	Odds ratio^a^	95% CI	*p*-Value^a^
**MODEL 1 (HIGH hsCRP AND INDIVIDUAL CVD RISK FACTORS)**			
Central obesity (vs. no central obesity)	7.8	4.8–12.9	<0.001
Sex (male vs. female)	0.61	0.37–1.01	0.053
Parental education
Tertiary	Reference category		
Secondary	1.72	0.96–3.11	0.070
Primary/all age	2.47	1.17–5.20	0.017
Unknown	1.10	0.48–2.53	0.814
**MODEL 2 (HIGH HSCRP AND NUMBER OF METABOLIC SYNDROME COMPONENTS^b^)**			
Number of metabolic syndrome components
None	Reference category		
One component	2.2	1.3–3.8	0.005
Two components	7.4	4.1–13.6	<0.001
Three or more components (i.e., having the metabolic syndrome)	13.5	2.4–76.0	0.003
Sex (male vs. female)	0.5	0.3–0.9	0.011
Parental education
Tertiary	Reference category		
Secondary	1.8	1.0–3.2	0.051
Primary/all age	2.8	1.3–5.7	0.006
Unknown	1.4	0.6–3.0	0.463

*^a^Odds ratios and *p*-values were derived multivariable logistic model with high hsCRP as the outcome. Age was not significant and was therefore excluded from the model. Significance level adjusted to 0.025 based on Bonferroni correction for two independent hypotheses tested in the study*.

*^b^Risk factors included are elevated blood pressure, high fasting glucose, central obesity, low HDL, and high triglycerides. hsCRP, high sensitivity C-reactive protein; CVD, cardiovascular disease*.

## Discussion

In this study among young adults in Jamaica, we found that approximately 15% of participants had high risk hsCRP. High hsCRP was more common among women and was strongly associated with central obesity and the metabolic syndrome. There was also a strong association between high hsCRP and clustering of CVD risk factors, with higher odds of high hsCRP as the number of metabolic syndrome components increased. Additionally, there was a statistically significant trend for increasing prevalence of high hsCRP as the number of metabolic syndrome components increased. We also found that lower parental education was associated with higher odds of high hsCRP particularly among men.

The findings in this study are generally consistent with the published literature. Among CVD risk factors, the strongest associations appear to be with BMI and central obesity (31–34). In one study among adults 25–60 years old in Burkino Faso, central obesity was associated with a fourfold increase in the odds of high hsCRP (>1 mg/L) while overweight status was associated with an almost sevenfold increase in the odds in unadjusted models; however, in the fully adjusted model only central obesity and elevated triglyceride was associated with log hsCRP (32). In another study from China, while hsCRP showed significant correlations with all the CVD risk factors studied the largest correlation coefficients were seen for BMI and central obesity (31).

None of the other individual CVD risk factors studied were retained in the final model as associations were no longer significant after adjusting for confounding and model assessment indicated that they did not improve the model. These findings are consistent with results of the study from Burkino Faso and another study from the Netherlands where only central obesity and elevated triglyceride remained significant correlates of high hsCRP in multivariable models (32, 33). The absence of an association with elevated triglycerides in our study was probably due to the very low prevalence of hypertriglyceridemia (0.5%) in this study. It should be noted, however, that one study from Korea, however, reported a statistically significant inverse relationship between HDL and hsCRP (35).

A number of studies also reported strong associations between hsCRP and the number of CVD risk factors or metabolic syndrome components (31–33, 36, 37). In one study analyzing National Health and Nutrition Examination Survey (NHANES) 2009–10 data, participants with one metabolic syndrome component had a twofold higher odds of high hsCRP while participants with all five components had 11 times greater odds of high hsCRP (37). Being classified as having the metabolic syndromes was also associated with hsCRP level (16, 17, 31, 32). In the study from Burkino Faso, participants with hsCRP >1 mg/L were twice as likely to have the metabolic syndrome (32). Similarly, persons in the highest quartile of hsCRP were seven times more likely to have the metabolic syndrome compared to those in the lowest quartile (31).

We also found a significant inverse relationship between high hsCRP and parental education level, with the association being statistically significant for men whose highest parental education level was primary or all age (up to grade 9) when compared to those whose parents had tertiary level education. These findings are consistent with a growing body of literature showing associations between SES and hsCRP (13–15, 38–43). Most studies show an inverse association with higher levels of hsCRP with lower SES, with some studies showing significant sex-differences. For example, in a report from the Coronary Artery Risk Development in Young Adults (CARDIA) study, there were significant associations between CRP for White men and women and for Black women but not for Black men (42). Studies from Jamaica have generally shown sex-differences in the relationship between CVD and measures of SES (23, 44, 45). In a previous analysis from this cohort maternal occupation at time of child’s birth was found to be inversely related to BP, but was statistically significant in men only (46). The relationship between hsCRP and SES suggest that inflammation may be one of the mechanisms through which SES influence CVD risk, but this requires further study. We acknowledge that parental education as used here is a proxy for participants SES; however, parental SES was used since the participants were just entering into adulthood and at this age parental SES may be a better measure of SES than their own SES.

Prevalence of high hsCRP in this study was approximately 15%. There is little published data for this age group for comparison, however, one study reported a prevalence of 13% for elevated hsCRP (>3 mg/L) among 16-year-olds in Quebec, while another study using data from NHANES 2009–10 found that 32% of the US adult population had elevated hsCRP (19, 37). In another analysis from NHANES 2009–10, an estimated 29% of US adults 20–29 years old had hsCRP ≥3 mg/L (47). The mean and median hsCRP of our participants (1.5 and 0.7 mg/L, respectively) was lower than that of the 20- to 29-year-olds of the NHANES database 3.5 and 1.4 mg/L, respectively) (47).

It has been recognized that preventing or delaying the onset of the chronic non-communicable diseases is the goal of modern public health policy. To do this effectively requires early recognition of the occurrence of risk factors and taking action to promote optimal health in light of evidence suggesting that CVD risk factors tend to track from childhood into adulthood (48, 49). In young adulthood, many of the traditional risk factors are relatively uncommon and as such it may be difficult to predict those at highest risk using only traditional risk factors. In this study, while the prevalence of some individual risk factors was fairly high, <1% was classified as having the metabolic syndrome. If we used the metabolic syndrome as to classify participants with regards to future CVD risk, the large majority of participants would be classified as being at lower than average risk of CVD. The prevalence of high hsCRP, however, was approximately 15% suggesting that the many persons classified as low risk based on the metabolic syndrome criteria would be reclassified based on hsCRP. These findings suggest that hsCRP may be useful marker of CVD risk in young adults and may result in earlier identification of young adults at high risk for CVD.

This study had some limitations. Firstly, we acknowledge that there is significant short term variability hsCRP level, so measurement of hsCRP on a single occasion may result in some misclassification (5, 50). However, such misclassification is likely to be non-differential; therefore, the true associations are likely to be more extreme than reported. Additionally, there were missing data for some participants, while others had markedly elevated hsCRP, which resulted in their exclusion from the analyses. However, mean values for participant characteristics were similar for included and excluded participants, suggesting that this is unlikely to have influenced the findings of the study. Additionally, the consistency of our findings with other studies suggests that our estimates are plausible. Finally, the cross-sectional study design precludes any causal inferences from the study.

The study also had some important strength. Few studies have reported on hsCRP distribution among children, young adults, and in populations outside of Europe and North America (8). This has led to the Centers for Disease Control and American Heart Association specifically calling for studies among the young, the elderly, and within race/ethnic groups (8). This study was conducted among Afro-Caribbean youth in a developing country context and will be a valuable addition to the literature. Additionally, the study is population based and therefore findings are likely to be generalizable to urban Jamaica and to other populations with similar age and race/ethnic composition.

We conclude that high hsCRP is strongly associated with central obesity, the metabolic syndrome, and the number of cardiovascular risk factors. Our study suggests that hsCRP may improve CVD risk assessment in young adults when compared to the metabolic syndrome. Further longitudinal studies are needed to evaluate the predictive value of hsCRP in young populations. Additionally, the association between hsCRP and early markers of vascular health such as pulse wave velocity should be evaluated.

## Author Contributions

Nadia R. Bennett wrote first draft of manuscript, participated in data analysis and interpretation, critically revised manuscript. Trevor S. Ferguson supervised field activities for data collection, drafted proposal for sub-study, developed data-analysis plan, lead the data analysis, drafted revisions to manuscript, and critically reviewed the manuscript. Franklyn I. Bennett drafted proposal for sub-study; coordinated laboratory analyses; critically reviewed manuscript. Marshall K. Tulloch-Reid contributed to proposal for sub-study, contributed to data collection, interpretation of data analyses, and critical review of the manuscript. Novie O. M. Younger-Coleman contributed to data collection, data analysis, and critical review of the manuscript. Maria D. Jackson contributed to proposal for sub-study and interpretation of data analyses; critical review of the manuscript. Maureen E. Samms-Vaughan contributed to the design of the study and critical review of the manuscript. Rainford J. Wilks conceived and designed the study and directed its implementation; drafted proposal for sub-study; contributed to the data-analysis strategies and data interpretation; critically reviewed drafts of the manuscript.

## Conflict of Interest Statement

The authors declare that the research was conducted in the absence of any commercial or financial relationships that could be construed as a potential conflict of interest.

## Supplementary Material

The Supplementary Material for this article can be found online at http://www.frontiersin.org/Journal/10.3389/fcvm.2014.00012/abstract
