# Supplementary material for: High-Sensitivity C-Reactive Protein is Related to Central Obesity and the Number of Metabolic Syndrome Components in Jamaican Young Adults
Source: Front Cardiovasc Med. 2014 Dec 16;1:12. doi: 10.3389/fcvm.2014.00012 (PMC4668855; doi:10.3389/fcvm.2014.00012)
Supplement: Supplementary file 1 [file Data_Sheet_1.PDF]

High sensitivity C-reactive protein is related to central obesity and the number of metabolic syndrome components in Jamaican young adults

Nadia R Bennett[1], Trevor S Ferguson[1*], Franklyn I Bennett[2], Marshall K Tulloch-Reid[1], Novie OM Younger-Coleman[1], Maria D Jackson[3], Maureen Samms-Vaughan[4] Rainford J Wilks[1]

[1]Epidemiology Research Unit, Tropical Medicine Research Institute, The University of the West Indies Mona, Kingston, Jamaica
[2]Department of Pathology, The University of the West Indies Mona, Kingston, Jamaica
[3]Department of Community Health and Psychiatry, The University of the West Indies Mona, Kingston, Jamaica
[4]Department of Child and Adolescent Health, The University of the West Indies Mona, Kingston, Jamaica The University of the West Indies Mona, Kingston, Jamaica

Correspondence:

Dr. Trevor S Ferguson
Epidemiology Research Unit
Tropical Medicine Research Institute
The University of the West Indies,
Mona, Kingston 7
Jamaica
Email: trevor.ferguson02@uwimona.edu.jm
Telephone: 876 9272471
Fax: 876 9272984

SUPPLEMENTARY MATERIAL

Table S1: Proportion of participants with cardiovascular disease risk factors and high hsCRP for male and female participants

**Variables**	**Male**<br>n = 342<br>n (%)	**Female**<br>n = 404<br>n (%)	P-value for male: female differences[1]
**Elevated blood pressure**<br>(BP ≥120/80 mmHg)	**101 (29.5)**	53 (13.1)	**<0.001**
**Overweight**<br>(25-29.99 kg/m^2)	**47 (13.70**	79 (19.6)	**0.033**
**Obese**<br>(>=30 kg/m^2)	**17 (5.0)**	38 (9.4)	**0.018**
**High cholesterol**	**28 (8.2)**	76 (18.8)	**<0.001**
**Low HDL cholesterol**	**95 (27.8)**	248 (61.4)	**<0.001**
**High LDL cholesterol**	**10 (2.9)**	**38 (9.4)**	**<0.001**
**High triglycerides**	**2 (0.6)**	**2 (0.5)**	**0.868**
**High glucose**	**8 (2.3)**	**0 (0.0)**	**0.004**
**High WC**	**16 (4.7)**	**92 (22.8)**	**<0.001**
**High Risk hsCRP**	**29 (8.5)**	**81 (20.0)**	**<0.001**

[1]P-values are from proportion postestimation Wald tests

Table S2: Mean Values of Cardiovascular Disease Risk Factors by hsCRP categories for Male and Female Participants

Characteristic	Low risk[1] (*n*=235) Mean ± SD	Moderate risk[2] (*n*=78) Mean ± SD	High Risk[3] (*n*=43) Mean ± SD	p-value[4]
**MALES**				
**BMI**	21.8 ± 3.1	23.7 ± 3.9	26.3 ± 8.3	<0.001
**Waist circumference**	73.6 ± 9.7	77.3 ± 9.4	80.9 ± 18.8	<0.001
**Systolic blood pressure**	113.6 ± 10.6	115.0 ± 10.7	114.6 ± 11.4	0.556
**Diastolic blood pressure**	68.8 ± 10.0	69.8 ± 11.4	72.5 ± 8.5	0.166
**Fasting glucose**	4.7 ± 0.4	4.7 ± 0.5	5.0 ± 1.3	0.630
**Fasting cholesterol**	4.1 ± 0.7	4.2 ± 0.9	4.0 ± 0.8	0.670
**Fasting HDL**	1.2 ± 0.2	1.1 ± 0.2	1.1 ± 0.3	0.145
**Fasting LDL**	2.7 ± 0.6	2.8 ± 0.8	2.7 ± 0.7	0.509
**Fasting triglycerides**	0.6 ± 0.3	0.6 ± 0.2	0.6 ± 0.3	0.478
**FEMALES**				
**BMI**	20.9 ± 3.1	24.0 ± 4.4	27.4 ± 7.0	<0.001
**Waist circumference**	68.7 ± 7.1	75.1 ± 9.4	82.7 ± 14.3	<0.001
**Systolic blood pressure**	105.9 ± 8.0	108.6 ± 9.5	108.1 ± 8.6	0.013
**Diastolic blood pressure**	66.3 ± 8.4	68.2 ± 9.6	66.8 ± 9.2	0.172
**Fasting glucose**	4.4 ± 0.3	4.4 ± 0.4	4.5 ± 0.4	0.732
**Fasting cholesterol**	4.4 ± 0.8	4.7 ± 1.0	4.5 ± 0.9	0.225
**Fasting HDL**	1.3 ± 0.3	1.2 ± 0.3	1.2 ± 0.3	<0.001
**Fasting LDL**	2.9 ± 0.7	3.2 ± 1.0	3.0 ± 0.8	0.019
**Fasting triglycerides**	0.50 ± 0.2	0.61 ± 0.2	0.63 ± 0.3	<0.001

[1]Low risk = hsCRP <1mg/L; [2]Moderate risk hsCRP 1-3 mg/L; [3]High risk hsCRP >3mg/L
[4]P-values are from analysis of variance (ANOVA) F-test or non-parametric Kruskal-Wallis test (if there was evidence of unequal variance). Adjustment for multiple testing was not performed as these were considered preliminary exploratory analyses. If the Bonferroni correction for multiple testing were to be applied the p-value for statistical significance would be 0.006.
hsCRP = high sensitivity C-reactive protein; BMI = body mass index; HDL = high density lipoprotein cholesterol; LDL = low density lipoprotein cholesterol

Table S3: Mean and Median Value for hsCRP according to number of metabolic syndrome components among study participants

Number of Risk Factors*	Proportion of Participants n (%)	Mean hsCRP (mg/L)	Median hsCRP (mg/L)
None	348 (46.7)	1.0	0.5
One component	291 (39.0)	1.5	0.7
Two components	101 (13.5)	3.0	2.0
Three or more components (metabolic syndrome)	6 (0.8)	4.5	3.4

* Risk factors included are elevated blood pressure, high fasting glucose, central obesity, low HDL and high triglycerides using the standard metabolic syndrome cut points.
hsCRP = high sensitivity C-reactive protein; HDL = high density lipoprotein cholesterol
$p < 0.001$ for difference in means based of Kruskal-Wallis test; $p < 0.001$ for difference in medians; Non-parametric test for trend indicates p-value <0.001 for higher hsCRP as number of risk factors increase. Similar associations were seen in sex specific analyses

Table S4: Final sex-specific models for relationship between high hsCRP and individual CVD risk factors and number of metabolic syndrome components

Variable	Male			Female		
	Odds Ratio[1]	95 % CI	p-value[1]	Odds Ratio[1]	95 % CI	p-value[1]
Model 1 (High hsCRP and individual CVD Risk Factors)						
**Central Obesity** (vs. no central obesity)	**7.8**	2.3 - 26.7	0.001	**8.3**	4.8 - 14.4	<0.001
**Parental Education**						
**Tertiary**	**Reference**	**Reference**	**Reference**	**Reference**	**Reference**	**Reference**
**Secondary**	**1.5**	0.5 - 4.6	0.444	**1.8**	0.9 - 3.7	0.096
**Primary/All Age**	**6.0**	1.7 - 21.5	0.006	**1.6**	0.0 - 3.9	0.335
**Unknown**	**0.3**	0.03 - 2.3	0.228	**1.8**	0.7 - 4.7	0.259
Model 2 (High hsCRP and Number of Metabolic Syndrome Components[2])						
**Number of Metabolic Syndrome Components**						
**None**	**Reference**	**Reference**	**Reference**	**Reference**	**Reference**	**Reference**
**One component**	**1.62**	0.66 - 3.98	0.295	**3.19**	1.47 - 6.92	0.003
**Two components**	**3.78**	1.17 - 12.2	0.026	**11.7**	5.17 - 26.7	<0.001
**Three or more components**	**24.4**	1.23 - 485.9	0.036	**12.3**	1.52 - 99.3	0.019
**Parental Education**						
**Tertiary**	**Reference**	**Reference**	**Reference**	**Reference**	**Reference**	**Reference**
**Secondary**	**1.66**	0.53 - 5.35	0.376	**1.86**	0.93 - 3.71	0.077
**Primary/All Age**	**6.25**	1.68 - 12.2	0.06	**1.86**	0.76 - 4.53	0.172
**Unknown**	**0.30**	0.03 - 2.85	0.299	**2.27**	0.87 - 5.92	0.091

[1]Odds ratios and P-values are derived from multivariable logistic regression models. Sex specific models shown here as there was evidence of sex interaction in the relationship between high hsCRP and education level (p = 0.049 for the unknown education category, but not significant for the other categories). Significance level adjusted to 0.025 based on Bonferroni correction for two independent hypotheses tested in the study.
[2]Risk factors included are elevated blood pressure, high fasting glucose, central obesity, low HDL and high triglycerides; persons with three or more components would be classified as having the metabolic syndrome
hsCRP = high sensitivity C-reactive protein
